# pH/ROS-responsive smart hydrogels for infection control and immune microenvironment modulation in chronic wound healing

**DOI:** 10.3389/fcell.2026.1826583

**Published:** 2026-04-07

**Authors:** Rui Zhang, Suk Fei Tan, Wenqin Zhang, Ye Wang, Junxue Wu, Chao Zhang

**Affiliations:** 1 Department of Orthopedics, Affiliated Hospital of North Sichuan Medical College, Nanchong, Sichuan, China; 2 School of Graduate Studies, Post Graduate Centre, Management and Science University, Shah Alam, Malaysia; 3 School of Pharmacy, Management and Science University, Shah Alam, Malaysia; 4 Surgical Center, Affiliated Hospital of North Sichuan Medical College, Nanchong, Sichuan, China

**Keywords:** chronic wound, diabetic wound, immune microenvironment, infection control, macrophage polarization, pH-responsive, ROS-responsive, smart hydrogel

## Abstract

Chronic wounds are characterized by persistent infection, excessive oxidative stress, and dysregulated inflammation, which together hinder tissue repair and promote non-healing states. In this context, pH/ROS-responsive hydrogels have emerged as promising smart wound-dressing platforms because they can exploit local acidic and oxidative cues for site-specific structural change, controlled cargo release, and microenvironment-adaptive therapy. This review summarizes the pathological basis for pH/ROS-responsive intervention and discusses recent advances in hydrogel design for infection control, ROS scavenging, and immune microenvironment modulation. We further highlight key translational challenges, including manufacturing complexity, degradation-product safety, storage stability, and the limitations of current preclinical models. Overall, pH/ROS-responsive hydrogels represent a promising strategy for chronic wound management, but future progress will depend on achieving a better balance between multifunctional performance and translational practicality.

## 1 Introduction

Chronic wounds are a major clinical and socioeconomic burden, especially in patients with diabetes, vascular insufficiency, aging, and immunometabolic dysfunction. Unlike acute wounds, chronic wounds are locked in a state of unresolved inflammation and are frequently accompanied by bacterial persistence, oxidative stress, defective angiogenesis, and impaired matrix remodeling (Hartmeier et al., 2021; Louiselle et al., 2021; Chen et al., 2023; Fan et al., 2024). Among them, diabetic wounds are particularly challenging because hyperglycemia, hypoxia, ischemia, neuropathy, and recurrent infection jointly generate a hostile local microenvironment (Louiselle et al., 2021; Sharifiaghdam et al., 2022).

**FIGURE 1:**
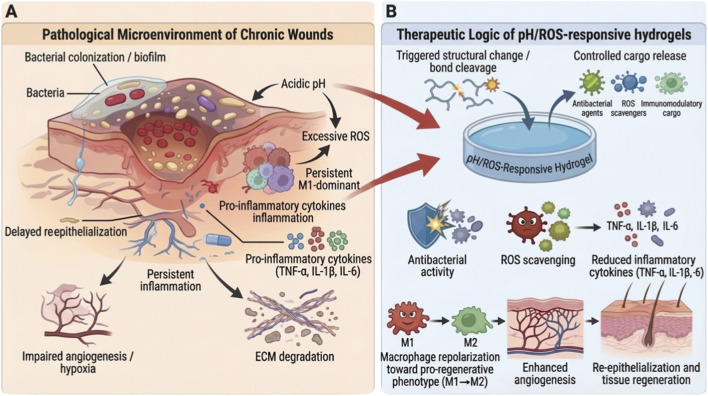
Pathological microenvironment of chronic wounds and therapeutic logic of pH/ROS-responsive hydrogels. **(A)** Chronic wounds are characterized by bacterial colonization or biofilm formation, acidic pH, excessive reactive oxygen species (ROS), persistent M1-dominant inflammation, elevated pro-inflammatory cytokines (TNF-α, IL-1β, and IL-6), extracellular matrix (ECM) degradation, impaired angiogenesis/hypoxia, and delayed re-epithelialization. These pathological features interact to maintain a self-sustaining non-healing wound microenvironment. **(B)** pH/ROS-responsive hydrogels exploit acidic and oxidative cues as endogenous triggers for structural change, bond cleavage, and controlled cargo release. Through antibacterial activity, ROS scavenging, inflammatory attenuation, macrophage repolarization toward a pro-regenerative phenotype, enhanced angiogenesis, and tissue regeneration, these systems help restore a repair-permissive wound microenvironment. This figure is a conceptual schematic rather than an experimental workflow.

Hydrogels have become one of the most promising advanced wound-dressing platforms because their hydrated three-dimensional networks resemble extracellular matrix, maintain moisture, absorb exudate, and can be engineered for injectability, adhesion, self-healing, and controlled release (Liu and Ge, 2025; Ma et al., 2025). More importantly, recent hydrogel research has moved beyond passive coverage toward dynamic microenvironment regulation. Smart hydrogels responsive to endogenous wound cues, such as pH, ROS, glucose, enzymes, and inflammatory mediators, are especially attractive for chronic wound treatment (Das and Bal, 2024; Fan et al., 2024; Liu and Ge, 2025). As illustrated in Figure 1A, chronic wounds exhibit a self-perpetuating pathological microenvironment characterized by bacterial colonization or biofilm formation, acidic pH, excessive ROS, persistent inflammation, extracellular matrix degradation, impaired angiogenesis, and delayed re-epithelialization.

Recent studies further support the importance of microenvironment-centered hydrogel therapy in chronic wounds. For example, immunoregulatory hydrogels capable of oxygenation and ROS neutralization have shown promise in diabetic foot ulcer repair, reinforcing the therapeutic significance of oxidative stress control and inflammatory reprogramming in chronic wound healing (Qi et al., 2025). In parallel, intrinsically antibacterial hydrogel systems have demonstrated that effective infection control can be combined with macrophage phenotype transition, collagen deposition, and neovascularization, highlighting the therapeutic coupling between antimicrobial action and regenerative immune modulation (Cheng et al., 2022).

Among these systems, pH/ROS-responsive hydrogels are particularly relevant because infected chronic wounds commonly exhibit local acidosis and excessive oxidative stress. Acidification is associated with bacterial metabolism, inflammatory exudate, and impaired tissue buffering, whereas ROS overproduction arises from prolonged neutrophil and macrophage activation (Li et al., 2022). These two cues therefore represent biologically meaningful endogenous triggers for site-specific structural transformation, controlled cargo release, and microenvironment-responsive therapy (Das and Bal, 2024; Deng et al., 2024).

Unlike broader reviews on smart wound dressings or general responsive hydrogels, the present review specifically focuses on pH/ROS-responsive hydrogels as pathology-matched platforms for chronic wounds. We emphasize their dual therapeutic logic in infection control and immune microenvironment modulation, while also critically discussing translational bottlenecks, including scale-up feasibility, degradation-product safety, storage stability, and the limitations of current preclinical validation systems. This perspective aims to bridge material design with clinically relevant therapeutic and translational considerations.

## 2 Pathological basis for pH/ROS-responsive intervention

The chronic wound niche is shaped by several interconnected abnormalities. First, persistent bacterial colonization and biofilm formation reduce antibiotic sensitivity and impair host clearance (Hartmeier et al., 2021; Huang et al., 2024). Second, excessive ROS damages keratinocytes, fibroblasts, endothelial cells, and extracellular matrix, thereby delaying granulation, angiogenesis, and re-epithelialization (Chen et al., 2023; Wang P. et al., 2024). Third, immune dysregulation maintains macrophages in an M1-dominant state, with sustained production of TNF-α, IL-1β, IL-6, and proteolytic enzymes, preventing the transition toward tissue repair (Li et al., 2022; Tu et al., 2022). As summarized in Figure 1B, pH/ROS-responsive hydrogels exploit acidic and oxidative cues as endogenous triggers for structural transformation, controlled cargo release, antibacterial action, ROS scavenging, immune microenvironment modulation, and regenerative repair.

pH-responsive hydrogel systems take advantage of the acidic microenvironment of infected wounds through dynamic chemistries such as Schiff-base, acylhydrazone, and boronate ester bonds. These mechanisms allow selective swelling, partial degradation, and drug release under acidic conditions (Das and Bal, 2024; Deng et al., 2024). ROS-responsive systems use oxidant-sensitive linkages, including thioketal, phenylboronic ester, selenium-containing motifs, and catechol-related oxidation pathways, enabling cargo release or direct ROS neutralization in oxidative wound niches (Das and Bal, 2024; Wang W. et al., 2024). Integration of both pH and ROS responsiveness helps match hydrogel function to real pathological fluctuations in chronic wounds.

## 3 pH/ROS-responsive hydrogels for infection control

Infection control is the first therapeutic priority in chronic wounds. Responsive hydrogels improve local anti-infective therapy in several ways. They can release antibiotics, antimicrobial peptides, metal-polyphenol complexes, or bioactive phytochemicals specifically in acidic and oxidative wound sites, thereby reducing off-target exposure and increasing local bioavailability (He et al., 2024; Wang W. et al., 2024). In addition, hydrogel matrix adaptation may improve contact with irregular wound beds and support biofilm disruption.

Recent studies increasingly combine anti-infective treatment with oxidative stress regulation and inflammatory remodeling instead of addressing bacterial burden alone. Deng et al. reported a microenvironment-responsive smart hydrogel with antibacterial activity and immune-regulatory capability for chronic wound repair, illustrating the value of integrating infection control with microenvironment remodeling (Deng et al., 2024). Triple-responsive hyaluronic acid systems, multifunctional photothermal hydrogels, and pH-probe-integrated infected-wound dressings have further expanded this concept into “sense-and-treat” platforms (He et al., 2024; Huang et al., 2024; Xiang et al., 2024).

Notably, next-generation infection-responsive hydrogels are no longer restricted to classic antibiotic delivery. Nanozyme-based dressings can combine antibacterial activity with catalytic oxygen generation and ROS balancing, thereby addressing infection, hypoxia, and oxidative damage simultaneously (Deng et al., 2023; Zhao et al., 2023). Biomass-derived and peptide-based responsive hydrogels similarly show potential for long-term bacterial suppression while preserving tissue compatibility (Wu et al., 2024; Ma et al., 2025). These multifunctional anti-infective platforms are particularly suitable for chronic wounds, where infection, inflammation, and regeneration failure are tightly coupled.

## 4 Immune microenvironment modulation

Successful healing of chronic wounds requires more than bacterial eradication. The immune microenvironment must also be reprogrammed from persistent inflammation toward controlled resolution and regeneration. Macrophages are central to this transition. M1 macrophages support pathogen clearance in early inflammation, whereas M2 macrophages promote angiogenesis, collagen deposition, and extracellular matrix remodeling (Louiselle et al., 2021; Li et al., 2022; Tu et al., 2022). In diabetic and infected wounds, however, this phenotypic switch is often blocked.

pH/ROS-responsive hydrogels can reshape this imbalance through several mechanisms. ROS-scavenging modules reduce oxidative injury and dampen inflammatory signaling (Deng et al., 2023; Zhao et al., 2023). Responsive delivery of exosomes, cytokines, growth factors, nucleic acids, or small molecules can promote macrophage repolarization toward M2-like phenotypes (Zeng et al., 2023; Shu et al., 2024; Xu et al., 2024; He et al., 2025; Kuan et al., 2025). Oxygen-regulating systems further alleviate hypoxia, which is closely associated with persistent inflammation and endothelial dysfunction (Deng et al., 2023; Zhao et al., 2023).

This dual-action strategy—combining anti-infective efficacy with immunomodulation—is one of the strongest advantages of pH/ROS-responsive hydrogels. For example, immunomodulatory hydrogels capable of orchestrating macrophage pro-regenerative behavior and promoting angiogenesis have shown promising performance in chronic wound models (Tang et al., 2025; Zhang et al., 2025). MMP-responsive and exosome-releasing systems also suggest that future hydrogels may provide staged and pathology-matched treatment rather than one-time bulk release (He et al., 2025; Kim et al., 2025; Meng et al., 2025). Thus, the key significance of pH/ROS-responsive hydrogels lies not merely in “smart release,” but in smart release aligned with wound immunopathology.

## 5 Representative design strategies

**FIGURE 2:**
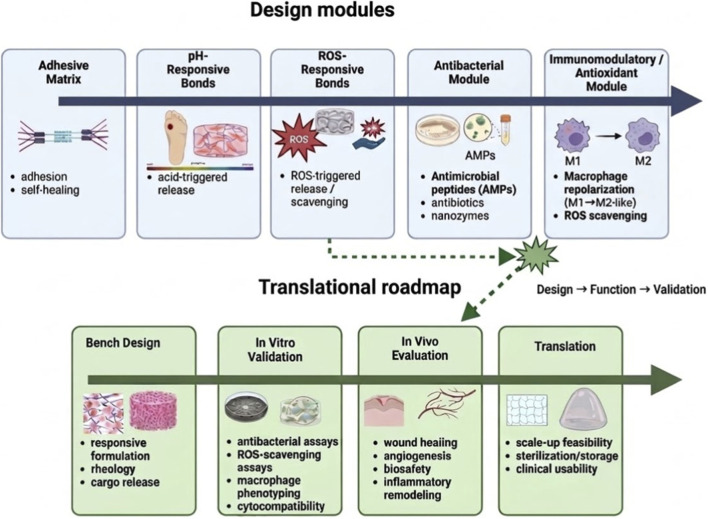
Design modules and translational roadmap of pH/ROS-responsive hydrogels for chronic wound healing. The upper panel summarizes the principal design modules of pH/ROS-responsive hydrogels, including adhesive matrices, pH-responsive bonds, ROS-responsive bonds, antibacterial modules, and immunomodulatory/antioxidant modules. These design elements support key therapeutic functions such as wet adhesion, stimulus-triggered release, infection control, oxidative-stress attenuation, and macrophage-centered immune regulation. The lower panel outlines a simplified translational roadmap from bench design to *in vitro* validation, *in vivo* evaluation, and practical translation, highlighting representative considerations including rheological behavior, cargo release, antibacterial and ROS-scavenging assays, macrophage phenotyping, cytocompatibility, biosafety, inflammatory remodeling, scale-up feasibility, sterilization/storage, and clinical usability. This figure is a conceptual framework rather than a standardized regulatory pathway.

The design of pH/ROS-responsive smart hydrogels generally follows a pathology-matched strategy, in which responsive matrices, dynamic bonds, antibacterial components, and immunomodulatory or ROS-scavenging modules are integrated into multifunctional systems. As summarized in Figure 2, the design of pH/ROS-responsive hydrogels for chronic wound healing typically integrates adhesive matrices, stimulus-responsive bonds, antibacterial modules, and immunomodulatory/antioxidant components to achieve coordinated therapeutic functions.

### 5.1 Dynamic covalent adhesive hydrogels

These systems commonly use boronate ester, Schiff-base, catechol, or acylhydrazone chemistry to achieve wet adhesion, injectability, self-healing, and biochemical responsiveness. They are well suited to moist, irregular, and exudative wound beds (Das and Bal, 2024; Deng et al., 2024).

### 5.2 ROS-scavenging and oxygen-regulating hydrogels

These hydrogels integrate antioxidant polyphenols, catalytic nanoparticles, or nanozymes to consume excess ROS and, in some cases, generate oxygen *in situ*. Such designs are attractive because oxidative stress and tissue hypoxia often coexist in diabetic wounds (Deng et al., 2023; Zhao et al., 2023).

### 5.3 Immunomodulatory cargo-loaded hydrogels

These platforms deliver exosomes, miRNAs, cytokines, or small molecules to repolarize macrophages and restore regenerative signaling. Several recent studies have shown improved wound closure and angiogenesis using exosome-loaded or immune-guided hydrogel systems (Zeng et al., 2023; Xu et al., 2024; Meng et al., 2025).

### 5.4 Integrated theranostic hydrogels

These platforms combine wound-state monitoring, such as pH sensing, with responsive treatment. Although still early in translation, they may become important for precision wound management by providing visual or measurable feedback linked to treatment response (He et al., 2024).

Although these design strategies highlight the versatility of pH/ROS-responsive hydrogel platforms, their functional sophistication also introduces practical constraints. Responsive chemistries that perform well under controlled laboratory conditions may show greater vulnerability to precursor variability, storage instability, sterilization-related structural alteration, or uncertain degradation-product safety during translation. Therefore, evaluation of these systems should consider not only therapeutic efficacy, but also manufacturability, reproducibility, and post-preparation stability.

## 6 Challenges and future perspectives

Despite rapid progress, several barriers continue to limit the clinical translation of pH/ROS-responsive smart hydrogels. A major issue is that many high-performance systems are chemically sophisticated but structurally complex, which may improve responsiveness in laboratory settings while simultaneously reducing process simplicity, batch reproducibility, sterilization compatibility, and scale-up feasibility (Maity et al., 2025). Dynamic covalent strategies based on Schiff-base, acylhydrazone, boronate ester, or catechol-related interactions are attractive because they support injectability, self-healing, wet adhesion, and stimulus-responsiveness (Xia et al., 2026); however, their translational suitability is unlikely to be equivalent. Systems requiring multistep synthesis, stringent precursor control, nanoparticle integration, or multi-cargo encapsulation may face substantial manufacturing and quality-control barriers despite promising biological performance (Aldana et al., 2021). The lower panel of Figure 2 outlines a simplified translational roadmap for pH/ROS-responsive hydrogels, spanning bench design, *in vitro* validation, *in vivo* evaluation, and practical translation.

Another underappreciated challenge is the biosafety of matrices, cargos, and degradation products. Many studies primarily report short-term cytocompatibility or wound closure outcomes, while providing limited information on the local and systemic fate of cleavage byproducts generated from reversible dynamic bonds or ROS-responsive linkages (Zhang et al., 2024). This concern is particularly relevant for chemically responsive motifs such as boronate ester-containing systems, oxidant-sensitive moieties, and nanozyme-integrated platforms, where degradation products, ion release, or long-term tissue exposure may affect inflammatory signaling, hemocompatibility, or metabolic clearance (Liu et al., 2020). Future studies should therefore move beyond general statements of “good biocompatibility” and include more rigorous evaluation of degradation-product toxicity, immunotoxicity, hemocompatibility, and long-term biosafety.

Storage and transport stability also deserve greater attention. Highly sensitive linkages may enhance pathological responsiveness but can also increase the risk of premature hydrolysis, oxidation, structural relaxation, or loss of function during storage and handling (Terriac et al., 2024). For example, reversible pH-sensitive or boronate ester-based networks may be susceptible to ambient moisture, temperature fluctuation, oxidative exposure, or prolonged storage, potentially compromising mechanical integrity, drug-retention behavior, and on-demand responsiveness. From a translational perspective, shelf-life, packaging strategy, sterilization tolerance, and post-storage performance retention are as important as freshly prepared *in vitro* responsiveness (Lu et al., 2026).

A further limitation is that most current evidence still derives from rodent wound models, which do not fully recapitulate the size, biomechanics, microbial diversity, host comorbidity burden, and chronic inflammatory persistence of human chronic wounds. In particular, diabetic or infected wounds in humans often involve biofilm complexity, ischemia, recurrent contamination, and prolonged dressing-management challenges that are difficult to model in short-duration small-animal studies (Cirka and Nguyen, 2025). Future validation should therefore incorporate more clinically relevant models, including infected diabetic wound models with biofilm burden, longer observation windows, and, where feasible, large-animal platforms. Outcome assessment should also be expanded beyond wound closure to include bacterial burden, oxidative stress markers, macrophage phenotype, angiogenesis, matrix remodeling, scar quality, and recurrence risk.

Looking forward, the most translatable pH/ROS-responsive hydrogels may not be the most chemically elaborate ones, but rather those that achieve a practical balance among responsiveness, safety, mechanical adaptability, manufacturability, and clinical usability. Simplified but multifunctional systems, stage-adaptive release strategies, and standardized translational evaluation frameworks may help move this field from proof-of-concept innovation toward clinically actionable wound therapy.

## 7 Conclusion

pH/ROS-responsive smart hydrogels represent a promising class of next-generation wound dressings because they directly engage the defining pathologies of chronic wounds: infection, oxidative stress, and immune dysregulation. Their greatest strength lies in coupling responsive drug delivery with active microenvironment remodeling, particularly macrophage-centered immunomodulation. With further improvements in biosafety, manufacturability, and translational validation, these biomaterials may help move chronic wound care from passive dressing replacement toward precise, pathology-guided intervention.
